# Effect of Huaier on Melanoma Invasion, Metastasis, and Angiogenesis

**DOI:** 10.1155/2020/8163839

**Published:** 2020-06-07

**Authors:** Dongqiang Su, Bingbing Jiang, Yun Yang, Yu Miao, Qian Fu, Feng Zhang

**Affiliations:** Department of Dermatology, First Affiliated Hospital of Harbin Medical University, Harbin 150001, China

## Abstract

Malignant melanoma (MM) is a highly metastatic and malignant cancer. Developing potential drugs with good efficacy and low toxicity for MM treatment is needed. Huaier, extracted from the mushroom *Trametes robiniophila* Murr, has been widely used in clinical anticancer treatments in China. A previous work done by our group confirmed that Huaier could inhibit cell proliferation and induce apoptosis in human melanoma cells. The current study is aimed at investigating the effects of Huaier on melanoma metastasis and angiogenesis *in vitro* and *in vivo* and to explore its possible mechanism of action. Our results showed that Huaier not only significantly inhibited the metastasis of A375 cells at the concentration ranging from 4 to 16 mg/ml (*P* < 0.05), which were determined by the wound healing assay and transwell assay *in vitro*, but also suppressed the MM tumor growth and metastatic cells to the liver in A375-bearing mice after oral administration at the dose of 5 mg/kg (*P* < 0.05). In addition, Huaier treatment downregulated the expression of hypoxia-inducible factor-1*α* (HIF-1*α*), vascular endothelial growth factor (VEGF), astrocyte-elevated gene-1 (AEG-1), and N-cadherin, while it upregulated E-cadherin expression in both the A375 cells and tumor tissues, which was detected using western blotting and RT-PCR (*P* < 0.05). Taken together, our data suggests that the antitumor and antimetastatic activities of Huaier are caused by the downregulation of the HIF-1*α*/VEGF and AEG-1 signaling pathways and by the inhibition of epithelial-mesenchymal transition (EMT). This study provides a new insight into the mechanism of Huaier on antimetastatic therapy and a new scientific basis for comprehensive treatment strategies for MM.

## 1. Introduction

Melanoma is a malignant tumor that originates from melanocytes [1]. Despite advances in the treatment of melanoma, MM has a very poor prognosis with invasion and metastasis remaining the leading cause of death [2]. Treatments with fewer adverse effects, improving the quality of life, and maximizing survival are an important part of the treatment of malignant metastatic melanoma [3]. Traditional Chinese medicine (TCM) has been used in China for many years and has many advantages such as low price, overall conditioning, and fewer adverse effects. It plays an important role in the treatment of malignant tumors and has been reported to enhance the sensitivity of the tumor to radiotherapy and chemotherapy, alleviate adverse reactions [4], and cause an inhibitory effect on tumor invasion and metastasis [5]. The pharmacological effects and pharmaceutical ingredients of many TCMs are constantly being studied, deepening the understanding of TCMs' action mechanisms.

*Trametes robiniophila* Murr (Huaier) is a fungus found in China that has been used in TCM for approximately 1600 years [6]. Huaier has been shown not only to inhibit the proliferation of melanoma cells and other solid tumors but also to promote tumor cell apoptosis and inhibit tumor cell invasion, metastasis, and angiogenesis in many types of solid tumors such as hepatocellular carcinoma and colorectal carcinoma [6–9]. Previous studies in our group have shown that Huaier can inhibit the proliferation of melanoma cells and promote apoptosis [9].

However, antiproliferative and proapoptosis activities are not sufficient to explain the effects of Huaier in cancer treatment; an antimetastatic activity may be an important contributor. *In vitro* and *in vivo* studies have shown that Huaier can inhibit the invasiveness and metastasis of human hepatocellular carcinomas [10, 11]. Further research on the antimetastasis and anti-invasion effects of Huaier in melanoma is needed. In this context, we attempted to evaluate whether Huaier can inhibit angiogenesis and suppress tumor growth and metastasis in MM. The aim of this study is to provide a theoretical basis for Huaier as an adjuvant treatment for melanoma.

## 2. Materials and Methods

### 2.1. Materials and Chemicals

Huaier extract was donated by Qidong Gai Tianli Pharmaceutical Co., Ltd. (Jiangsu Province, China). Huaier extract 1 g was dissolved in 10 ml complete medium and sterilized with a 0.22 mm filter to obtain a 100 mg/ml stock solution and stored at −20°C. Fresh dilutions of the medium were prepared in each experiment. Fetal bovine serum (FBS) was purchased from Gibco (San Diego, USA). Hematoxylin and eosin was obtained from Solarbio (Beijing, China) and Sangon (Shanghai, China), respectively. Antibodies against VEGF, HIF-1*α*, AEG-1, E-cadherin, N-cadherin, and *β*-actin were obtained from Wanleibio (Shenyang, China). Matrigel for the transwell experiments was purchased from Becton, Dickinson and Company (New Jersey, USA). RNase inhibitor, TRIzol reagent, and the cDNA Transcription Kit were all obtained from BioTeke (Beijing, China). SYBR Green was obtained from Solarbio (Beijing, China). The microscope used was an Olympus inverted microscope CKX41 (Olympus, Tokyo, Japan). All other chemicals were of the highest commercial grade.

The human melanoma A375 cell line was obtained from the Beirui Biotechnology Co., Ltd. (Nanjing, China). The cell line was cultured in Dulbecco's Modified Eagle Medium (DMEM) medium with 10% FBS at 37°C in a humidified atmosphere of 5% CO_2_. Male BALB/c nude mice, aged 4 to 6 weeks and weighing 15-22 g, were purchased from Beijing Vital River Laboratory Animal Technology Co., Ltd. (China). All animals had free access to food and water under specific pathogen-free (SPF) conditions and were maintained in a pathogen-free environment at 25 ± 2°C and humidity of 55% ± 5% under a 12/12 h light/dark cycle. The Ethics Committee of Harbin Medical University approved all experimental protocols described in this study.

### 2.2. Wound Healing Assay

A375 cells were seeded in 6-well plates. After reaching confluency, a scratch was made on the surface of the wells with a 200 *μ*l tip. The cells were then incubated with serum-free medium containing different concentrations of Huaier (0, 4, 8, and 16 mg/ml, respectively). At 24, 48, and 72 h of incubation, cell migration was observed and photographs were taken under an inverted microscope. The distances between the edges of the simulated wound were also compared.

### 2.3. Transwell Assay

A 50 *μ*l mixture of Matrigel, which was dissolved in DMEM at a ratio of 1 : 11, was gently added to the upper chamber of a transwell system. 1 × 10^5^ cells were added into the upper chamber, together with various concentrations of Huaier (0, 4, 8, or 16 mg/ml). After 24 h incubation at 37°C, the cells that migrated to the lower surface of the filters were fixed with ethanol and stained with 1% crystal violet. The stained cells were counted using an inverted microscope. Above is the procedure of the invasion assay. The migration assay was performed in the same manner as the invasion assay, except for the upper chamber not being coated with Matrigel. After an 8 h incubation period, the experiment was terminated and the results were recorded.

### 2.4. Western Blotting Analysis

A375 cells were routinely cultured up to 50% confluence in 25 cm^2^ cell culture flasks and incubated with Huaier at 0, 4, 8, and 16 mg/ml for 48 h. The proteins harvested from the cells were lysed in a lysis buffer. Subsequently, the proteins from each sample were separated with 10% sodium dodecyl sulfate polyacrylamide gel electrophoresis (SDS-PAGE) and electrotransferred onto polyvinylidene fluoride (PVDF) membranes. After blocking with 5% nonfat milk, the PVDF membranes were covered with specific primary antibodies against-E-cadherin, N-cadherin, VEGF, AEG-1, HIF-1*α*, and *β*-actin, followed by incubation with secondary antibodies. Finally, the protein bands were visualized with an enhanced chemiluminescence (ECL) system. The density of the luminescence was analyzed on the Gel-Pro-Analyzer System (Beijing Liuyi Biotechnology Co., Ltd., Beijing, China).

### 2.5. *In Vivo* Antitumor Assay

A diluted A375 cells suspension (0.2 ml, 1 × 107 cells/mouse) was injected subcutaneously into the left arm pit of the nude BALB/c mice at day 0. Mice (14 in total) were randomly divided into a Huaier-treated group and nontreated control group with 7 mice in each group. On the second day after modeling the mice, Huaier (5 mg/kg) or normal saline were administered intragastrically once a day for 28 consecutive days. The size of the solid tumor was measured every 3 days until the day of sacrifice. After measuring the width and length of each tumor, size was calculated using Equation (1):

(1) $$\begin{array}{c} \text{Tumor volume }\left({\text{mm}}^{3}\right)=\left({\text{width}}^{2}\times \text{length}\right)/2 \end{array}$$

After 24 h following final administration (day 28), mice were sacrificed. The whole body and local tumors were measured immediately. The inhibition rate (IR) of tumor growth was calculated and compared with those of the control group by using Equation (2):

(2) $$\begin{array}{c} I\text{R }\left(\%\right)=\left[\left(\text{Vc}-\text{Vt}\right)/\text{Vc}\right]\times 100 \end{array}$$

Vc represents the average tumor weight of the control group, and Vt that of the treatment group. A small piece from each tumor was fixed in 4% buffered, freshly prepared paraformaldehyde, embedded in paraffin, sectioned into paraffin sections, deparaffinized, and stained with hematoxylin and eosin.

### 2.6. *In Vivo* Antiliver Metastasis Assay

A375 cells were suspended in 200 *μ*l of phosphate buffered saline (PBS) and injected into BALB/c nude mice via the tail vein (5 × 106 cells for each mouse). To study the development of liver metastasis formation, the mice were grouped and treated as described previously on day 2. Treatment also lasted for 28 days, after which the mice were sacrificed by cervical dislocation and the livers removed for liver metastasis examination. The tumor metastasis in liver tissue was observed under a microscope and immunohistochemical analysis was performed.

### 2.7. Immunohistochemistry of Mouse Liver

Liver tissues were fixed in 10% formalin, embedded in paraffin, and sliced into 4 *μ*m sections. Endogenous peroxidase activity was inhibited with 3% hydrogen peroxide (H_2_ O_2_) for 15 min. After incubation with anti-HMB45 and anti-S100 antibodies at 4°C overnight, the sections were washed and treated with biotinylated anti-immunoglobulin antibody for 20 min and then reacted with horseradish peroxidase-conjugated streptavidin. Thereafter, diaminobenzidine (DAB) was used, followed by counterstaining with hematoxylin. The representative images of tumor tissues were taken by an Olympus light microscope.

### 2.8. Real-Time PCR

Real-time PCR was used to detect mRNA levels of E-cadherin, N-cadherin, VEGF, HIF-1*α*, and AEG-1 in tumor tissues of tumor-bearing mice. Total RNA was isolated from tumor tissue using the TRIzol reagent (BioTeke, Beijing, China). The RNA was reverse transcribed into cDNA using the cDNA Reverse Transcription Kit according to the manufacturer's instructions (BioTeke, Beijing, China). 1 *μ*l of the resulting cDNA was used as a template for real-time PCR using SYBR Green (Solarbio, Beijing, China). RNA levels were quantified using the 2^-*ΔΔ*CT^ method. The two groups were compared for statistical differences in gene expression. The primer sequences used are shown in Table 1.

### 2.9. Statistical Analysis

Data acquired were processed using SPSS 20.0 software. *P* < 0.05 was considered statistically significant.

## 3. Results

### 3.1. Results of the Wound Healing Assay

**Figure 1:**
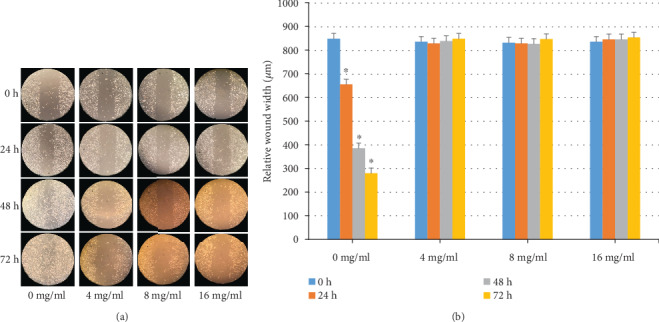
The wound healing assay. Melanoma A375 cells were treated with different concentrations of Huaier (0, 4, 8, and 16 mg/ml) and observed at 24, 48, and 72 h (a). At 24, 48, and 72 h (b), the healing status of the 4, 8, and 16 mg/ml Huaier-treated groups was significantly different compared to the control group (0 mg/ml) (^∗^*P* < 0.05, respectively; magnification ×100).

The migration distances between the leading edges of the simulated wound lines were compared between Huaier-treated and control cells. As shown in Figure 1, the migration capacity of A375 cells decreased after Huaier treatment. The wound width of the control group and Huaier-treated group was similar at 0 h after scratching. However, after 24, 48, and 72 h, the wound width of the Huaier groups was significantly wider than that of the control group (*P* < 0.05).

### 3.2. Results of the Transwell Assay

**Figure 2:**
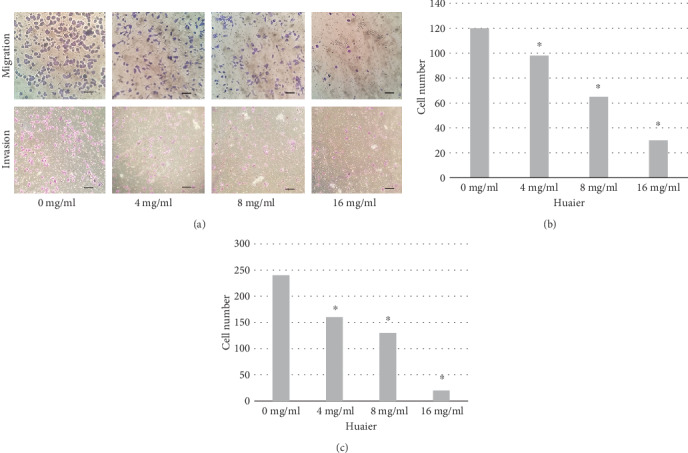
Transwell migration assay to examine the effect of Huaier on the migration and invasion of melanoma A375. A375 cells (a) were treated with different concentrations of Huaier (0, 4, 8, and 16 mg/ml). The incubation time of the invasion experiment was 24 hours and the migration experiment was 8 hours. Quantitative analysis of Huaier-induced inhibition of migration (b) and invasion (c) of A375 cells *in vitro*. With the increasing concentration of Huaier, the number of migrating or invading cells passing through the chamber gradually decreased in a concentration-dependent manner, with a significant difference when compared to the control group (0 mg/ml; ^∗^*P* < 0.05, respectively). Black bars represent 200 *μ*m.

As shown in Figure 2, after treatment with different concentrations of Huaier (4, 8, and 16 mg/ml), the number of migrating and invading cells that successfully passed through the membrane (Figures 2(b) and 2(c), respectively) was markedly reduced in a concentration-dependent manner compared with that of the control group (*P* < 0.05).

### 3.3. Results of Western Blotting Analysis of Huaier on AEG-1, VEGF, HIF-1*α*, E-Cadherin, and N-Cadherin Protein Expression in A375 Cell

**Figure 3:**
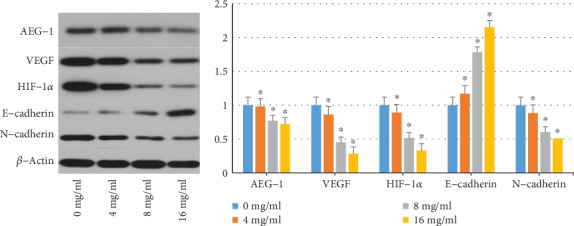
Effect of Huaier on the expression of AEG-1, VEGF, HIF-1*α*, and EMT signaling pathways in A375 cells.

We examined a series of protumor mediators associated with angiogenesis and metastasis including AEG-1, VEGF, HIF-1*α*, E-cadherin, and N-cadherin to explore the potential mechanism by which Huaier reduces angiogenesis and metastasis in A375 cells. As shown in Figure 3, following the exposure to Huaier for 48 h, the expression levels decreased (and increased for E-cadherin) in a dose-dependent manner and was significantly different when compared to the untreated control group. This data suggests the capacity of Huaier to suppress tumor angiogenesis and metastasis *in vitro* might be caused by the downregulation of HIF-1*α*, VEGF, and AEG-1 protein expression and reversing EMT.

After treatment with the various concentrations of Huaier (4, 8, and 16 mg/ml) for 48 h, the expression level of E-cadherin protein increased in a concentration-dependent manner. The expression levels of AEG-1, VEGF, HIF-1*α*, and N-cadherin gradually decreased in a concentration-dependent manner. Results were statistically significant when compared with the control group (0 mg/ml; ^∗^*P* < 0.05, respectively).

### 3.4. Effect of Huaier on Tumor Growth in Nude Mice

**Figure 4:**
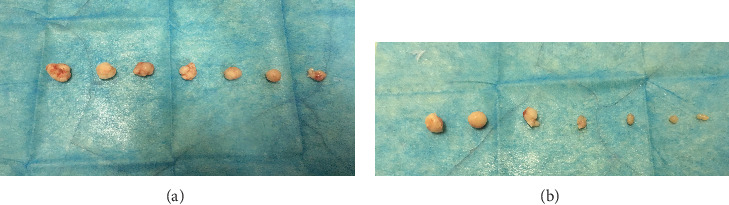
Metastatic tumor of the underarm of tumor-bearing mice. (a) Control group. (b) The Huaier-treated group. The mean tumor volume of the Huaier-treated group was smaller than the control group (*P* < 0.05).

**Figure 5:**
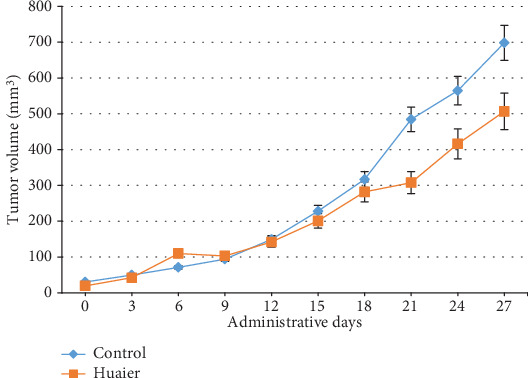
Relative mean tumor volume of the mice in the different groups. The blue line represents the control group while the orange line represents the Huaier-treated group. From the 21st day, the mean tumor volume in the control group was larger than that in the Huaier-treated group (*P* < 0.05). Data is presented as mean ± SD (*n* = 7).

The *in vivo* antitumor effect of Huaier was evaluated using the implanted A375 xenograft mouse model. Tumor sizes were measured using a caliper every three days until the end of the experiment to monitor the *in vivo* therapeutic efficiency. As shown in Figures 4 and 5, the mean tumor size of the control mice maintained a higher growth rate compared to the Huaier-treated mice. There was no significant difference in tumor volume between the two groups during the early stages of the experiment; however, the difference became significant after the 21st day. At the end of the study, the mean volume of the treatment group and the control group were 503.46 ± 45.13 mm^3^ and 698.40 ± 87.96 mm^3^, respectively (*P* < 0.05; Table 2). In addition, during this period, mice from both groups had a normal appetite and there was no significant difference in body weight between the two groups.

Data are expressed as mean ± SD (*n* = 7); ∗*P* < 0.05 compared with the untreated control group.

### 3.5. Effect of Huaier on Tumor Metastasis in Nude Mice Tested by Immunohistochemistry

**Figure 6:**
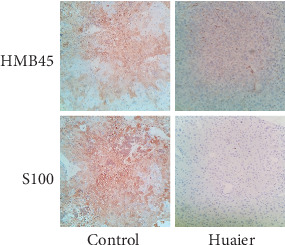
Immunohistochemical analyses of melanoma markers in the liver tissue of mice. Expression of HMB45 and S100 markers monitored by immunohistochemistry in the liver tissue of mice. The expression of HMB45 and S100 in the Huaier-treated group was lower compared to the control group (*P* < 0.05; magnification ×100).

Microscopic observation revealed more tumor cells in the control group compared to the Huaier-treated group. Immunohistochemical staining for S-100, HMB-45 was performed on the liver of the model mice (Figure 6). Positive expression of S-100 and HMB-45 of melanoma was revealed with brown or yellow particles appearing in the membrane or cytoplasm. The control group showed strong S-100 and HMB-45 positive expression compared to the statistically significant weak positive expression observed in the Huaier-treated group (*P* < 0.05).

### 3.6. Effect of Huaier on AEG-1, VEGF, HIF-1*α*, E-Cadherin, and N-Cadherin mRNA Expression in Nude Mice Tested by Real-Time PCR

**Figure 7:**
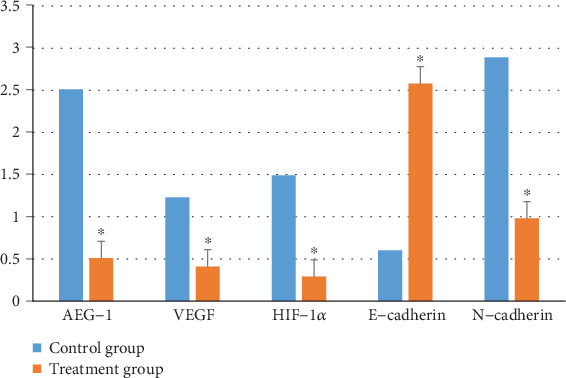
Effect of Huaier on the mRNA expression of AEG-1, VEGF, HIF-1*α*, and EMT signaling pathways in tumor. Compared to the control group, the expression level of E-cadherin increased, while the expression levels of AEG-1, VEGF, HIF-1*α*, and N-cadherin decreased (^∗^*P* < 0.05, respectively).

As shown in Figure 7, the mRNA expression of AEG-1, VEGF, HIF-1*α*, and N-cadherin was downregulated while the expression of E-cadherin was reversed in the Huaier-treated group. The difference was statistically significant when compared to the control group (*P* < 0.05). These results corroborated the *in vitro* cell results described above.

## 4. Discussion

Huaier has been widely used in China for many years. As far as we know, there are few studies on the inhibitory effect of Huaier on melanoma metastasis. In this study, the effects of Huaier on invasion, metastasis, and angiogenesis of melanoma cells were demonstrated *in vitro* and *in vivo*.

The wound healing and transwell assay results demonstrated that Huaier effectively attenuated the migration and invasion abilities of A375 cells. *In vivo* experiments illustrated that Huaier could significantly inhibit tumor metastasis and reduce liver metastasis in mice.

Recent studies have validated the association between the invasion and metastasis potential of cancer cells and the activation of EMT [12]. EMT is marked by decreased expression of E-cadherin and increased expression of N-cadherin. The suppression of EMT is emerging as a common mechanism underlying the inhibitory effect on the metastasis potential of cancer cells [13]. Some studies have found that Huaier can inhibit liver cancer and gastric cancer by inhibiting the EMT pathway [14, 15]. In this study, Huaier was found to significantly reduce the EMT process in A375 cells by reducing the expression of E-cadherin and increasing the expression of N-cadherin. These findings suggest that Huaier may inhibit invasion and metastasis by reversing the EMT process of melanoma cells. Tumor angiogenesis is a physiological process required for tumor metastasis. HIF-1*α* and VEGF are important angiogenic factors, and their elevated productions are related to tumor angiogenesis and tumor development [16]. The HIF-1*α*/VEGF signaling pathway has been demonstrated to play a role in the progression of melanoma metastasis [17]. In addition, it has been shown that HIF-1*α* can induce EMT in many types of cancer tissues. VEGF is the key downstream effector of HIF-1*α* and plays a key role in inducing cell migration and tube formation [18]. Recent studies have shown that Huaier can inhibit liver cancer by downregulating the expression of HIF-1*α* and VEGF [14]. The *in vitro* and *in vivo* results in this study showed that Huaier decreased the expression of HIF-1*α* and VEGF in melanoma tissues. This suggests that Huaier can play a role in the prevention of MM by inhibiting angiogenesis and blocking the tumor metastasis pathway.

AEG-1 has emerged as an essential oncogene in regulating multiple aspects of cancer development and progression, including metastasis. It is important for tumor growth and cell migration and invasion mediated by EMT [19]. Increasing evidence shows that the level of AEG-1 is elevated in MM and that silencing of AEG-1 significantly inhibits the proliferation and metastasis of melanoma cells [20]. It has been shown that Huaier can inhibit the growth and metastasis potential of hepatoma cells by regulating the AEG-1/EMT pathway [21]. We, therefore, analyzed the AEG-1 protein expression in A375 cells in response to Huaier treatment. The expression of AEG-1 was significantly decreased in Huaier-treated cells and tumors, compared to the untreated cells and tumors. We can thus conclude that Huaier may exert partial anti-invasive and antimetastatic effects by inhibiting AEG-1.

Invasion and metastasis are the major causes of death in melanoma patients. Palliative treatment of patients who have been transferred is trying to stop or delay the progress of the disease, and prolonging their life is an important part of the treatment of melanoma. Huaier is natural, nontoxic, and cheap and is exhibiting tumor growth and metastatic inhibition properties. We propose that Huaier possesses a high therapeutic potential against metastatic melanoma cells and could be used as an effective supplement for patients with other traditional Chinese medicines or other therapeutic methods. Further investigation regarding its mechanism of action and clinical trials are warranted.

Future research will focus on further investigation of the action mechanism pathways of Huaier and doing additional clinical research in melanoma patients.

## 5. Conclusions

The experiment illustrated the ability of Huaier to inhibit the migration and invasion of melanoma A375 cells in a concentration-dependent manner by reversing the EMT process and downregulating the AEG-1 pathway. Results also indicated that Huaier can inhibit melanoma invasion and tumor metastasis. By downregulating the activity of the HIF-1*α*/VEGF signaling pathway, Huaier may have an effect on angiogenesis of melanoma.

## Figures and Tables

**Table 1:** The primer sequences.

HIF-1*α* forward	AGTGTACCCTAACTAGCCG
HIF-1*α* reverse	CACAAATCAGCACCAAGC
VEGF forward	GACAAGGCAGACTATTCAG
VEGF reverse	CTCTTGATACCTCTTTCGTCT
AEG-1 forward	GTTGAAGTGGCTGAGGGT
AEG-1 reverse	CATGGCGTGAACTGTTTT
E-cadherin forward	TCAAAGTGGCGACAGACGG
E-cadherin reverse	GTTGGATTCAGAGGCAGGGT
N-cadherin forward	ATCCTACTGGACGGTTCG
N-cadherin reverse	AGTTGACTGAGGCGGGTG
*β*-Actin forward	CTGTGCCCATCTACGAGGGCTAT
*β*-Actin reverse	TTTGATGTCACGCACGATTTCC

**Table 2:** Effect of Huaier on body weight and tumor volume in nude mice bearing human melanoma A375 cells.

Group	Doses (mg/kg/day)	Body weight(g)		Tumor volume (mm^3^)	IR(%)
		Beginning	End		
Control (normal saline)	5	22.96 ± 5.02	23.70 ± 4.83	698.40 ± 87.96	—
Huaier	5	22.76 ± 3.86	23.13 ± 3.81	503.46 ± 45.13∗	27.9

## Data Availability

The data used to support the findings of this study are included within the article, and the original data used to support the findings of this study are available from the corresponding author upon request.
